# Sigma-1 Receptor as a Protective Factor for Diabetes-Associated Cognitive Dysfunction via Regulating Astrocytic Endoplasmic Reticulum-Mitochondrion Contact and Endoplasmic Reticulum Stress

**DOI:** 10.3390/cells12010197

**Published:** 2023-01-03

**Authors:** Mengyu Du, Tao Jiang, Shuxuan He, Bo Cheng, Xin Zhang, Liya Li, Lan Yang, Wei Gao, Yansong Li, Qiang Wang

**Affiliations:** 1 Department of Anesthesiology & Center for Brain Science, The First Affiliated Hospital of Xi’an Jiaotong University, Xi’an 710061, China; 2 Department of Anesthesiology, The Second Affiliated Hospital of Xi’an Jiaotong University, Xi’an 710004, China; 3 Department of Anesthesiology, The Second Affiliated Hospital of Dalian Medical University, Dalian 116023, China

**Keywords:** complement component 3, diabetes-associated cognitive dysfunction, endoplasmic reticulum stress, mitochondria-associated endoplasmic reticulum membrane, sigma-1 receptor, synapse deficits, type 1 diabetes mellitus

## Abstract

The prevalence of diabetes-associated cognitive dysfunction (DACD) has increased to 13.5%. Dementia, as the most severe DACD, is the second leading cause of death in patients with diabetes mellitus. Hence, the potential mechanisms of DACD for slowing or halting its progression need to be urgently explored. Given that the sigma-1 receptor (Sig-1R), a chaperone protein located in the endoplasmic reticulum (ER)-mitochondrion contact membranes to regulate ER stress (ERS), is associated with cognitive outcomes in neurodegenerative diseases, this study aimed to investigate the role of astrocytic Sig-1R in DACD and its underlying mechanism. Here, we examined the levels of ERS and complement component 3/3a (C3/C3a) from primary astrocytes with different concentrations of glucose and treatment. Subsequently, HT22 neurons were cultured in different astrocyte-conditioned medium, and the expression of synaptic proteins was detected. We constructed type 1 diabetes mellitus (T1DM) model to evaluate the astrocytic Sig-1R mechanism on synapse and cognitive function changes. In vitro, high glucose concentration downregulated Sig-1R and aggravated ERS in astrocytes, resulting in synapse deficits. PRE-084, a high-affinity and selective Sig-1R agonist, inhibited astrocytic ERS and complement cascades and restored synaptic damage, while the Sig-1R antagonist displayed the opposite results. Moreover, C3a receptor antagonist (C3aRA) could mimic the effect of PRE-084 and exerted neuroprotective effects. In vivo, PRE-084 substantially reduced ER-mitochondrion contact, activation of ERS, and C3/C3a secretion in mice with T1DM. Additionally, the synaptic loss and neurobehavioral dysfunction of mice with T1DM were less pronounced in both the PRE-084 and C3aRA treatment groups. These findings demonstrated that Sig-1R activation reduced the astrocytic ER-mitochondrion contact, ERS activation, and complement-mediated synaptic damage in T1DM. This study suggested the mechanisms and potential therapeutic approaches for treating DACD.

## 1. Introduction

The prevalence of diabetes-associated cognitive dysfunction (DACD) has increased to 13.5% [1]. As the most severe DACD, dementia has evolved as the secondary contributor to death in patients with diabetes [2]. Although type 1 diabetes mellitus (T1DM) is less common than T2DM, its incidence has increased worldwide. The new cases of T1DM in China are predicted to increase by 1.57 times over the next decade [3]. Patients with T1DM, especially the young ones, confer a higher risk of subsequent dementia than those with T2DM [4]. Therefore, the underlying mechanisms of DACD, particularly T1DM-related cognitive decline, need to be explored in further studies.

Endoplasmic reticulum stress (ERS), as a universal mechanism that participates in many neurodegenerative diseases [5,6] and chronic metabolic diseases such as diabetes [7], results in misfolded and unfolded protein accumulation and disruption of regular endoplasmic reticulum (ER) functions. The sigma-1 receptor (Sig-1R), located in mitochondria-associated endoplasmic reticulum membrane (MAM), usually binds to binding immunoglobulin protein (BIP/GRP78) as an ER chaperone to ensure the function and stability of certain signaling molecules, regulating calcium homeostasis, improving MAM function, and preventing the occurrence of ERS [8,9]. It is broadly spread throughout the brain, including the hippocampus, and is implicated in cytodifferentiation, neuroprotection, neuroplasticity, and cognitive function [10]. Sig-1Rs are enriched in neurons, astrocytes, and microglia [11]. Although neuronal Sig-1R has become a breakthrough target for alleviating neurodegenerative disorders, the effect of astrocytic Sig-1R is also significant since astrocytes also express it abundantly [12]. Sig-1R and its ligands may block the inflammatory response by decreasing the number of reactive astrocytes in rodent models of stroke and amyotrophic lateral sclerosis [13,14]. However, evidence confirming the Sig-1R changes in astrocytes in the brain of patients with diabetes is lacking; hence, further studies are needed to elucidate the cellular mechanisms of astrocytic Sig-1R during DACD.

Complement component 3 (C3), the core molecule in the complement cascade, contributes to a markedly increased risk of developing diabetes [15,16] and various neurodegenerative diseases [17]. Following the cleavage of C3 into C3a and C3b, C3a can bind to the C3a receptor (C3aR) to elicit downstream events [18,19]. Through down-regulation of C3 in vitro, LPS-induced lung cells were able to maintain viability and suppress apoptosis by decreasing ERS expression [20]. In tunicamycin-established acute ERS models of piglets, the complement system was activated, which produced higher levels of activation product C3 [21]. Within the central nervous system, astrocytes are the primary source of the secreted C3, while microglia and neurons express C3aR [22]. Consistent with the previously reported destructive effect of the astrocytic nuclear factor κB (NF-κB)-activated C3 secretion on dendritic morphology and neuronal function [23,24], the upregulation of C3 played a pivotal role in driving synapse loss in the aging model [25] and several Alzheimer’s disease models [19,26]. Furthermore, the genetic ablation or deletion of C3 [27,28] and C3aR [18] recovered synaptic deficits and neuronal loss. Given that Sig-1R agonists could decrease ERS and protect neuronal structure and function in several neurodegenerative diseases, there might be a possible connection existed between Sig-1R-regulated ERS and C3 production. Nevertheless, evidence demonstrating that C3 contributes to DACD and Sig-1R regulates the generation of C3 is insufficient.

In this study, we demonstrated that activating astrocytic Sig-1R improved synaptic deficits and cognitive dysfunction in DACD, which involved reducing ER-mitochondrion contact, alleviating ERS, and decreasing C3/C3a production. These findings revealed the potential mechanism of Sig-1R in treating neurodegenerative diseases, which might be a potential therapeutic target for preventing DACD.

## 2. Materials and Methods

### 2.1. Cell Culture and Treatment

As described previously [23], the brains of 1–2-day-old mice were harvested for the preparation of primary astrocytes. After meningeal dissection, the cortex and hippocampus were isolated in Dulbecco’s modified Eagle’s medium (DMEM) (Gibco, Grand Island, NY, USA) and cut into pieces. The fragments were digested in 2.5% trypsin for 10 min at 37 °C. After adding a proper volume of DMEM containing 10% fetal bovine serum (FBS) (Gemini, Woodland, CA, USA) and blowing evenly using a glass dropper, the sample was centrifuged at 1000 rpm for 10 min. The cells were seeded into poly-l-lysine-coated (Solarbio, P8141, Beijing, China) 75-cm^2^ cell-culture flasks in DMEM containing 25 mmol·L^−1^ glucose, 1% penicillin/streptomycin (Gibco, USA), and 10% FBS with 95% air and 5% CO_2_ at 37 °C. After 7 days, the cells were shaken using a cell culture thermostatic oscillator and replating to remove the microglia. The culture medium was changed every 3–4 days. When cultured to 80% confluence, the astrocytes were incubated in serum-free DMEM containing 5.5 mmol·L^−1^ normal glucose (NG) or 55 mmol·L^−1^ high glucose (HG) concentration for 24-h experiments. For different concentrations of NG and HG, the astrocytes were treated with or without PRE-084 hydrochloride (an agonist of Sig-1R with high affinity and selectivity) (10 μmol·L^−1^, HY-18100A, MCE, Monmouth Junction, NJ, USA) or Sig-1R antagonist (Sig-1RA) (10 μmol·L^−1^, HY-10815, MCE, USA). Then, the control culture was mixed with the same concentration of dimethyl sulfoxide (DMSO). The cells treated with different conditions were named as NG, HG, HG + DMSO, HG + PRE-084, NG + DMSO, and NG + Sig-1RA groups. Astrocyte-conditioned medium (ACM) and cell proteins were harvested immediately for subsequent experiments.

Immortalized mouse HT22 hippocampal neurons (Beina Chuanglian Biotech Institute, Beijing, China) were cultured in DMEM (1% penicillin/streptomycin and 10% FBS with 5% CO_2_ at 37 °C) with the medium changed every 1–2 days. HT22 cell lines were extracted after replacing different ACM treatments for 24 h. The HT22 cells treated by different ACM were called NG, HG, HG + DMSO, HG + PRE-084, NG + DMSO, and NG + Sig-1RA groups. HT22 cells were incubated with HG-ACM, with a C3aR antagonist (C3aRA) (10 μmol·L^−1^, SB290157, Calbiochem, Billerica, MA, USA), or DMSO [19,24] to determine the role of C3a production in neuronal synapses. The HT22 cells were called HG + DMSO and HG + C3aRA groups, respectively.

### 2.2. Animal Participants and Drug Administration

In several groups throughout the experiments, healthy male C57BL/6 mice (8–10 weeks old; body weight 20–25 g) were purchased from Institute of Experimental Animals of the Xi’an Jiaotong University, Xi’an, China. The mice were kept in plastic cages having exhaust ventilation in central cages, with four mice per cage and a 12-h/12-h cycle, under a regulated living environment (room temperature 22.5 °C ± 1 °C and humidity 50% ± 5%). Standard laboratory food and water were provided to the mice. We attempted to minimize the number of animals and their suffering throughout the experiments. Animal studies were conducted strictly following the established guidelines of the Xi’an Jiaotong University’s Institute for Experimental Animals.

Mice were intraperitoneally injected with 50 mg/kg streptozotocin (STZ) (S0130, Sigma-Aldrich, St. Louis, MO, USA) dissolved in the sterile 0.1M citric acid buffer (pH = 4.5) for five consecutive days [29]. The control received the same volume of solvent injection. After 14 days of the STZ injections, the fasting blood glucose level was measured using blood glucose test strips (Yuwell Ltd., Danyang, Jiangsu, China). Mice with a blood glucose level above 16 mmol·L^−1^ were considered diabetic mice. Diabetic and healthy mice were randomly assigned to the following experimental groups: control (CON), CON + PRE-084, STZ, and STZ + PRE-084. Each group contained about 15 animals. Both PRE-084-treated groups received intraperitoneal PRE-084 solution at a dose of 0.25 mg/kg thrice per week from the age of 12–20 weeks [30]. The equivalent volume of sterile saline was injected into nontherapeutic mice. Moreover, we used C3aRA for a complementary test. The mice were randomly divided into the CON, CON + C3aRA, STZ, and STZ + C3aRA groups. Each group contained about 15 animals. Starting from the age of 17 weeks, the mice were treated with C3aRA (1 mg/kg) or phosphate-buffered saline (PBS) thrice a week via intraperitoneal injection for 3 weeks [19,24]. Given that mice with diabetes displayed behavioral cognitive dysfunction at the age of 20–26 weeks, as previously described [29], experiments were performed at least 12 weeks after STZ injection.

### 2.3. Western Blot Analysis

The cultured cells and hippocampal tissues were extracted using radioimmunoprecipitation assay buffer (P0013K, Beyotime, Shanghai, China) containing protease and phosphatase inhibitors (A32959, Thermo Fisher Scientific, Waltham, MA, USA), and centrifuged at 12,000 rpm and 4 °C for 10 min. The supernatant was harvested, and the proteins (20–30 μg of protein per lane) were run on 8–12% sodium dodecyl sulfate gel and then transferred to polyvinyl difluoride membranes. After being blocked with 5% skimmed milk for 1–2 h at room temperature, the membranes were incubated with specific antibodies: mouse anti-sigma receptor (1:100, sc-137075, Santa Cruz, CA, USA), rabbit anti-BIP (1:1000, 3177, Cell Signaling, Danvers, MA, USA), rabbit anti-p-PERK/anti-PERK (1:500, WL05295, Wanleibio, Shenyang, Liaoning, China/1:1000, 3192, Cell Signaling, USA), rabbit anti-p-eIF2α/anti-eIF2α (1:1000, 3398/5324, Cell Signaling, USA), rabbit anti-ATF4 (1:1000, WL02330, Wanleibio, China), rabbit anti-CHOP (1:500, WL00880, Wanleibio, China), rabbit anti-C3 (1:2000, ab200999, Abcam, Cambridge, MA, USA), mouse anti-PSD-95 (1:500, AF5283, Affinity, Cincinnati, OH, USA), mouse anti-Syp (1:500, AF0257, Affinity, USA), and rabbit anti-β-tubulin (1:2000, AC015, ABclonal, Wuhan, Hubei, China) overnight at 4 °C. The membranes were washed with Tris-buffered saline with 0.1% Tween 20 detergent and then incubated with corresponding horseradish peroxidase-labeled goat anti-rabbit/mouse IgG (1:5000, DY60202/DY60203, Diyibio, Shanghai, China) for 1–2 h at room temperature. The protein bands were visualized using an ECL kit (KF005, Affinity, USA). The intensity was measured using the ImageJ software (NIH, Bethesda, MD, USA) and corrected with the corresponding β-tubulin.

### 2.4. Enzyme-Linked Immunosorbent Assay Analysis

The supernatant of the primary astrocyte culture medium and hippocampal tissues of mice was collected, which was either immediately examined or stored at −80 °C for later analysis. The C3a secretory content was investigated using the mouse C3a enzyme-linked immunosorbent assay (ELISA) kits (JM-02685M1, Jingmeibio, Yancheng, Jiangsu, China), following the manufacturer’s protocols.

### 2.5. Transmission Electron Microscopy

Transmission electron microscope (TEM, Hitachi, Tokyo, Japan) was used to examine the ultrastructure of astrocytes and neurons. The tissues were fixed with 0.25% glutaraldehyde and 4% paraformaldehyde in 0.1M PBS (pH = 7.4). Subsequently, the isolated hippocampal tissues of CA1 area were placed into 2.5% glutaraldehyde at 4 °C for post-fixation. The hippocampal ultrastructure, especially the astrocytic MAM and synapses, was processed for observation using a TEM (Hitachi). The circumference of mitochondrion closely apposed to the ER (<30 nm) and the distance (<100 nm) were measured, as previously discussed [31,32]. Synapse deficits were identified by the changes in the synaptic cleft and the length and thickness of postsynaptic density (PSD) using the ImageJ software.

### 2.6. Golgi Staining

The brain tissues were separated and fixed in 4% paraformaldehyde for 24 h as previously reported with some modifications [33]. Then, the brain tissues were immersed in the 5% Golgi-Cox fixative solution, including potassium chromate, potassium dichromate, and mercury chloride. The coronal slices (100 μm thick) of brain tissues were made using a vibrating microtome for investigation. The images of the hippocampal CA1 region were captured using a super-resolution confocal microscope (Leica TCS SP8 STED 3X, Wetzlar, Germany). The Imaris software (v.9.7, Zurich, Switzerland) was used to reconstruct and calculate the spine density per 10 μm.

### 2.7. TUNEL Assay

After isoflurane inhalation anesthesia, the mice were perfused with 4% paraformaldehyde by cardiac perfusion and the brain was fixed in a 4% paraformaldehyde solution. The mouse brain was embedded in paraffin and cut into serial coronal sections. We strictly followed the manufacturer’s protocols for using terminal deoxynucleotidyl transferase-mediated deoxyuridine triphosphate (dTUP) nick-end labeling (TUNEL) assay kits (GDP1042, Servicebio, Wuhan, Hubei, China). The images of the hippocampal CA1 region were observed using Case Viewer C.V 2.3 and analyzed using the ImageJ software to count positive apoptotic cells.

### 2.8. Y-Maze Test

The Y maze consisted of three identical arms extending radially from the central equilateral triangle area to the surroundings with the 120° angle (each arm was 30 cm × 8 cm × 18 cm). The mice were placed at the intersection of the three arms for 8 min to explore freely. The mouse that entered all the three arms consecutively was recorded as a successful alternation. The accuracy of alternation was recorded as follows: spontaneous alternation behavior (%) = number of successful alternations/(total number of arm entries − 2) × 100%.

### 2.9. Morris Water Maze Test

The Morris water maze (MWM) test was performed to assess the memory ability of rodents for spatial learning. The maze was composed of a round black palace (diameter, 100 cm; height, 50 cm) filled with water (depth, 30 cm; temperature, 22 °C ± 1 °C) and a removable circular platform (10 cm in diameter) placed 1 cm below the water surface in the center of the fourth quadrant. Different visual signs of colors and shapes existed in the four quadrants around the palace. The underwater platform was hidden from mice by adding titanium dioxide to the water. The experiment consisted of two sessions. (1) The positioning of the training experiment lasted for five consecutive days, and the mice were trained via four trials a day from the four different quadrants of the apparatus with a certain intertrial interval. The escape latency (time to search for the submerged platform), swimming distance, and swimming speed were recorded automatically using the SMART 3.0 system with a camera for no more than 90 s. If the mouse could not locate the underwater platform, a 15-s on-platform stay was provided to them to learn how to access the platform. (2) The space exploration experiment was conducted on the sixth day. For this, the underwater platform was removed, and each mouse was placed in the second quadrant to explore for 90 s. The platform crossing times, target quadrant retention time, and distance were recorded as the indexes of spatial memory.

### 2.10. Statistical Analysis

Statistical analyses were performed using the GraphPad Prism 8.0 (La Jolla, CA, USA) software. All data were expressed as mean ± standard error of mean (SEM). Continuous data were analyzed using the Student’s *t* test, one-way analysis of variance (ANOVA) (followed by Tukey’s multiple comparison tests), Welch’s ANOVA (followed by Dunnett’s T3 multiple comparison tests), or Kruskal-Wallis one-way ANOVA (followed by Dunn’s multiple comparison tests) depending on the results of normal distribution and homogeneity of variance test. Two-way ANOVA was applied to analyze the MWM (group × day). A *p* value < 0.05 indicated a statistically significant difference.

## 3. Results

### 3.1. Decrease in Sig-1R Expression Was Associated with ERS of Astrocytes Exposed to a HG Concentration

**Figure 1:**
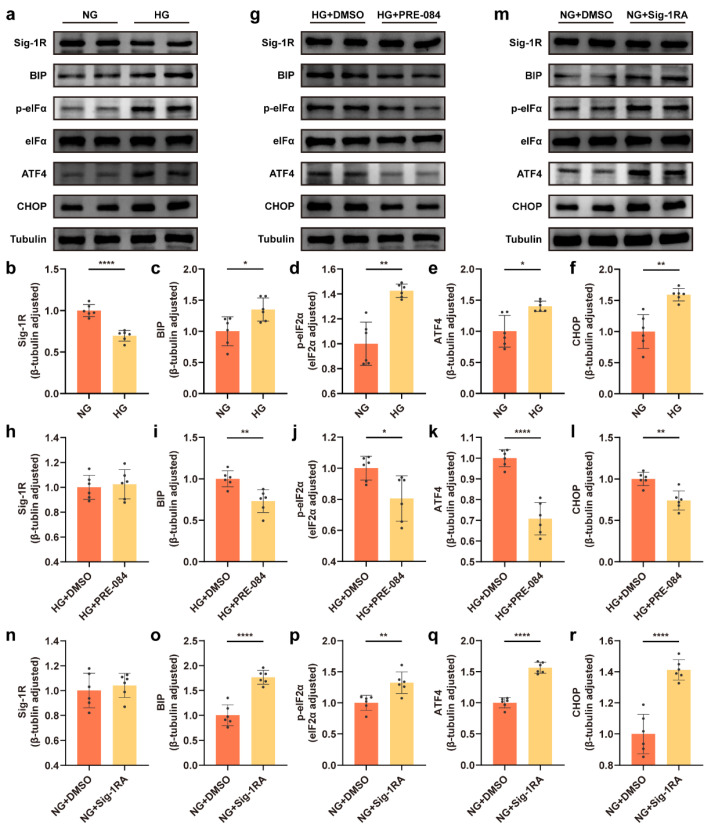
Sig-1R regulated HG-induced ERS in astrocytes. (**a**–**f**) Representative Western blot and quantitative analyses for Sig-1R and the ERS-related protein (BIP, p-eIF2α, eIF2α, ATF4, and CHOP) expression in the NG and HG groups (*n* = 6). (**g**–**l**) Representative Western blot images for the Sig-1R and ERS-related protein expression and corresponding quantitation in the HG + DMSO and HG + PRE-084 groups (*n* = 6). (**m**–**r**) Representative Western blot images and relevant quantitation of the Sig-1R and ERS-related protein expression in the NG + DMSO and NG + Sig-1RA groups (*n* = 6). The data were presented as mean ± SEM and analyzed using the Student’s *t* test or Welch’s *t* test. * *p* < 0.05; ** *p* < 0.01; **** *p* < 0.0001. Sig-1R, sigma-1 receptor; HG, high glucose; ERS, endoplasmic reticulum stress; BIP, binding immunoglobulin protein; eIF2α, eukaryotic translation initiation factor-2α; ATF4, activating transcription factor 4; CHOP, C/EBP homologous protein; NG, normal glucose; Sig-1RA, Sig-1R antagonist.

An in vitro model was developed to examine the effect of glucose concentrations (NG and HG) on the ERS in astrocytes using the Western blot technique. The results revealed that the expression of BIP protein significantly increased in the HG group (Figure 1a,c), and the dissociation of BIP from protein kinase RNA like ER-kinase (PERK) sensors induced ERS. In the HG group, the expression of other ERS-related proteins, including p-eukaryotic translation initiation factor-2α (eIF2α), activating transcription factor 4 (ATF4), and C/EBP homologous protein (CHOP) increased (Figure 1a,d–f). We treated the astrocytes with PRE-084 (a Sig-1R agonist with high selectivity) under HG incubation to investigate the role of activating Sig-1R in ERS of HG-induced astrocytes, and observed that BIP, p-eIF2α, ATF4, and CHOP protein levels were decreased in astrocytes in the HG + PRE-084 group compared with the HG-induced astrocytes (Figure 1g,i–l). Likewise, the reduced ERS-related proteins in the NG group were abolished after the treatment with Sig-1RA (a highly potent and selective Sig-1R antagonist) (Figure 1m,o–r). Interesting, Sig-1R, as an ER resident chaperone bound to BIP, considerably decreased in the HG group (Figure 1a,b), but PRE-084 and Sig-1RA had no influence on the expression of this protein (Figure 1g,h,m,n). These data verified that Sig-1R participated in HG-induced ERS in astrocytes.

### 3.2. Activation of Sig-1R Reduced ERS and Astrocytic ER-Mitochondrion Contact in the Hippocampus of Mice with T1DM

**Figure 2:**
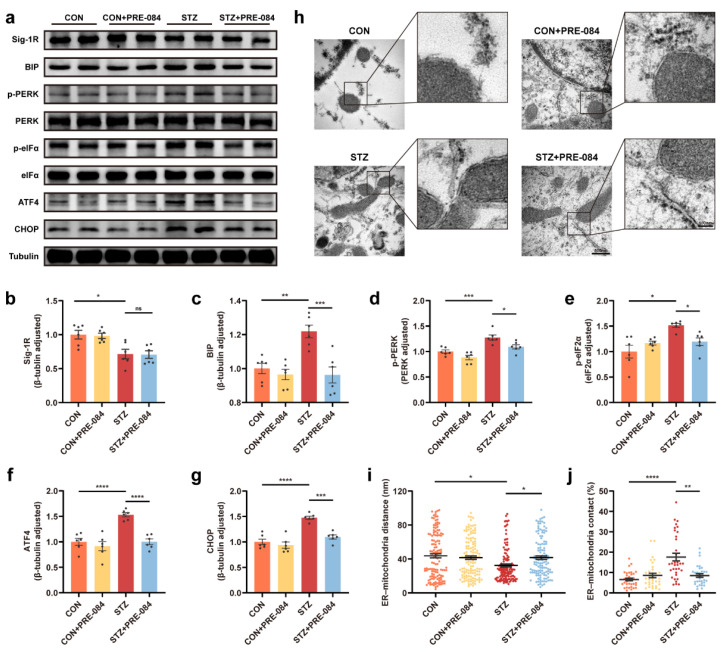
Sig-1R activation modulated ERS and astrocytic ER-mitochondrion contact in the hippocampus of mice with T1DM. (**a**–**g**) Representative Western blot and quantitative analyses of Sig-1R, BIP, p-PERK, PERK, p-eIF2α, eIF2α, ATF4, and CHOP protein expression in CON, CON + PRE-084, STZ, and STZ + PRE-084 mice (*n* = 6). (**h**) Representative electron micrographs of ER-mitochondria associations in astrocytes of the mouse hippocampus. The scale bars are 500 and 100 nm. (**i**,**j**) Quantitative analysis of the distance (*n* = 124–132; 3 mice per group) (**i**) and contact proportion (*n* = 32–34; 3 mice per group) (**j**) of ER-mitochondria associations. The data were presented as mean ± SEM and analyzed using one-way ANOVA with Tukey’s multiple comparisons test, Welch’s ANOVA test followed by Dunnett’s T3 multiple comparison tests, and Kruskal-Wallis test followed by Dunn’s multiple comparison tests. * *p* < 0.05; ** *p* < 0.01; *** *p* < 0.001; **** *p* < 0.0001. Sig-1R, sigma-1 receptor; ER, endoplasmic reticulum; ERS, ER stress; T1DM, type 1 diabetes mellitus; BIP, binding immunoglobulin protein; PERK, protein kinase RNA like ER-kinase; eIF2α, eukaryotic translation initiation factor-2α; ATF4, activating transcription factor 4; CHOP, C/EBP homologous protein; CON, control mice; STZ, mice with T1DM.

We used mice with STZ-induced T1DM to determine whether Sig-1R activation in DACD suppressed ERS in vivo. The results showed that the expression of Sig-1R decreased in the mice with T1DM, but had no significant difference compared with the mice with T1DM treated PRE-084 (Figure 2a,b). The Western blot revealed that the ERS-related protein expression was upregulated in the STZ group compared with the control group, and decreased in the mice with T1DM treated with PRE-084 (Figure 2a,c–g). Then, the proportion of the mitochondrial membrane that was close to the ER (<30 nm) and the distance between them in astrocytes were measured using TEM to examine the dynamic ER-mitochondria structures [31]. The increase in contact proportion corresponding to the decrease in distance between the mitochondrial membrane and ER in astrocytes was observed in the hippocampus of the T1DM model compared with that in the control brain, but PRE-084 treatment significantly reversed this trend (Figure 2h–j and Figure S1). The aforementioned findings revealed that Sig-1R agonist alleviated the activation of ERS and the enhancement of the ER-mitochondrion contact in astrocytes.

### 3.3. Activation of Sig-1R Ameliorated Synaptic Loss and Cognitive Dysfunction in Mice with T1DM

**Figure 3:**
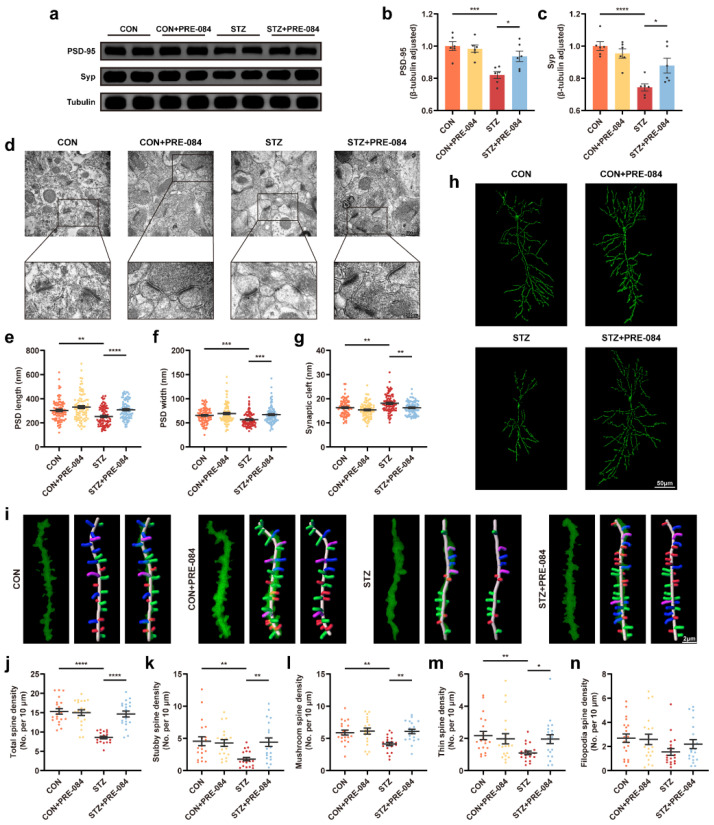
The effect of Sig-1R agonist on synapse change in the hippocampus of mice with T1DM. (**a**–**c**) Representative Western blots and quantitation of PSD-95 and Syp protein expression in CON, CON + PRE-084, STZ, and STZ + PRE-084 mice (*n* = 6). (**d**–**g**) Representative images of synaptic ultrastructure (**d**), quantitative analysis of PSD length (**e**) and width (**f**), and synaptic cleft (**g**) (*n* = 85–90; 3 animals per group). The scale bars are 500 and 200 nm. (**h**) Golgi staining images of the neuronal morphology. The scale bar is 50 μm. (**i**) Representative three-dimensional reconstruction images of different types of the spine in hippocampal neurons, including stubby (red), mushroom (green), thin (blue), and filopodia (purple) spines. The scale bar is 2 μm. (**j**–**n**) Quantitation of density of different spines, including total (**j**), stubby (**k**), mushroom (**l**), thin (**m**), and filopodia (**n**) spines (*n* = 20; 3 mice per group). The data were presented as mean ± SEM and analyzed via one-way ANOVA with Tukey’s multiple comparison tests, Welch’s ANOVA test followed by Dunnett’s T3 multiple comparison tests, and Kruskal-Wallis test followed by Dunn’s multiple comparison tests. * *p* < 0.05; ** *p* < 0.01; *** *p* < 0.001; **** *p* < 0.0001. Sig-1R, sigma-1 receptor; T1DM, type 1 diabetes mellitus; PSD, postsynaptic density; Syp, synaptophysin; CON, control mice; STZ, mice with T1DM.

**Figure 4:**
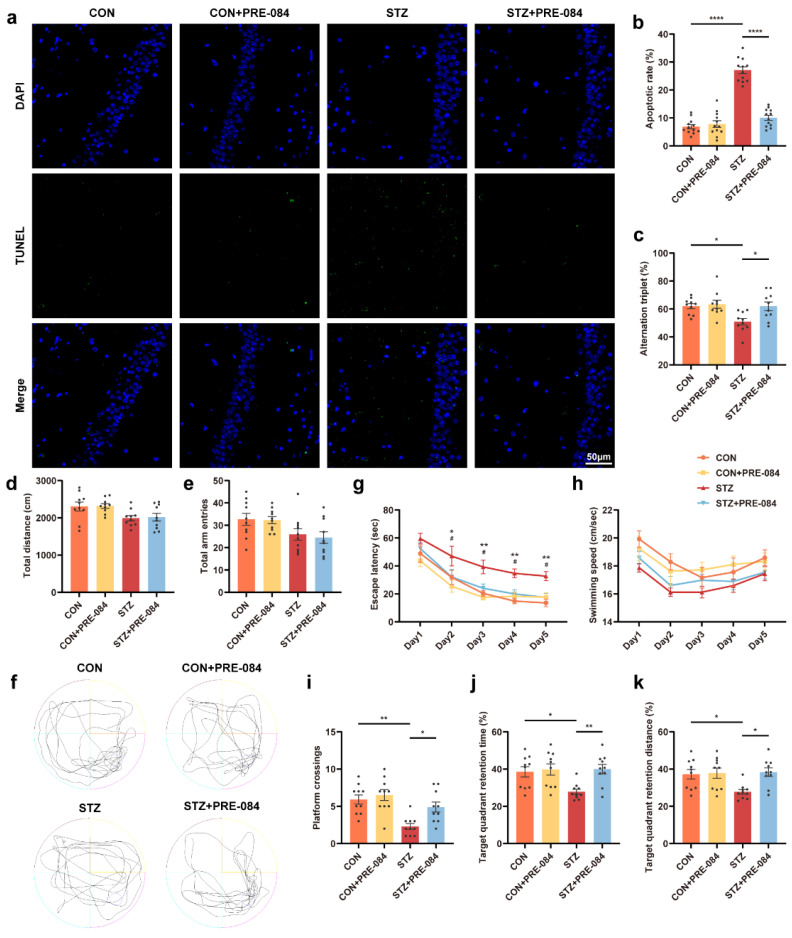
The effect of Sig-1R agonist on cognitive function in mice with T1DM. (**a**) Representative images of TUNEL labeling in the hippocampal CA1 region in the CON, CON + PRE-084, STZ, and STZ + PRE-084 groups. Green staining indicated TUNEL-positive cells, and blue staining indicated DAPI. The scale bar is 50 μm. (**b**) Quantitation of TUNEL-positive cells in the hippocampal areas (*n* = 12; 3 mice per group). (**c**–**e**) Y-maze alternation triplet (%) (**c**), total distance (**d**), and total arm entries (**e**) (*n* =10 mice for all groups). (**f**–**k**) Representative traces for MWM test (**f**) and the quantitation of escape latency (**g**), swimming speed (**h**), platform crossings (**i**), target quadrant retention time (**j**), and target quadrant retention distance (**k**) (*n* = 10) (* CON vs. STZ, ^#^ STZ vs. STZ + PRE-084). The data were presented as mean ± SEM and analyzed via one-way or two-way ANOVA with Tukey’s multiple comparison tests. * *p* < 0.05; ^#^ *p* < 0.05; ** *p* < 0.01; **** *p* < 0.0001. Sig-1R, sigma-1 receptor; T1DM, type 1 diabetes mellitus; TUNEL, terminal deoxynucleotidyl transferase-mediated dUTP nick-end labeling; CON, control mice; STZ, mice with T1DM; MWM, Morris water maze.

By Western blotting, we found that the protein levels of PSD-95 and synaptophysin (Syp) decreased in the mice with T1DM compared with the CON group, which were reversed after treatment with PRE-084 (Figure 3a–c). Furthermore, we visualized the synaptic structure in TEM. The images showed decreased PSD length and thickness coupled with a widened synaptic cleft in the hippocampus of mice with T1DM compared with that in the CON group, but the activation of Sig-1R distinctly reduced these values (Figure 3d–g). Based on Golgi staining, dendritic protrusions are typically divided into four categories, filopodia, thin, mushroom, and stubby spines. We found a varying reduction in different types of spines and a significant decrease in total dendritic spine density in the hippocampal CA1 region of mice with T1DM. The PRE-084 treatment effectively improved this condition (Figure 3i–n); however, no differences in density percentage of different types of spines were observed (Figure S2). Moreover, the hippocampal CA1 region neurons of mice with T1DM exhibited decreased neuronal complexity corresponding to shortened dendritic length. The neuronal complexity was elevated to a normal level after PRE-084 treatment, and the destructive dendritic length was repaired (Figure 3h). The immunofluorescence staining using TUNEL assay showed enhancement in cell apoptosis in the hippocampus of mice in the STZ group than in the CON group, which was significantly attenuated by PRE-084 treatment (Figure 4a,b). The Y-maze and MWM tests were performed to examine the behavioral cognitive function to assess the spatial learning memory ability of mice. No differences were observed in the total distance and total arm entry values in all groups (Figure 4d,e). However, the alternation triplet (%) in the Y-maze test was decreased in the STZ group. Treatment with PRE-084 significantly alleviated the impaired spontaneous activity (Figure 4c). As no difference was observed in the swimming speed between groups during the MWM test (Figure 4h), mice with T1DM exhibited meaningful extension of the escape latency compared with that in the control group during 2–5 days of positioning training experiment. The escape latency was almost shortened to normal in mice with T1DM in the PRE-084 treatment groups (Figure 4g). Further, the platform crossings, target quadrant retention time, and distance were decreased in the STZ group than in the CON group, whereas PRE-084 improved the spatial recognition disorder in mice with T1DM (Figure 4f,i–k). Based on the aforementioned findings, we concluded that the Sig-1R agonist could recover synaptic degeneration and cognitive disorder in mice with T1DM.

### 3.4. Sig-1R Regulation of C3/C3a Secretion Was Required for Controlled Synaptic Protein Loss

**Figure 5:**
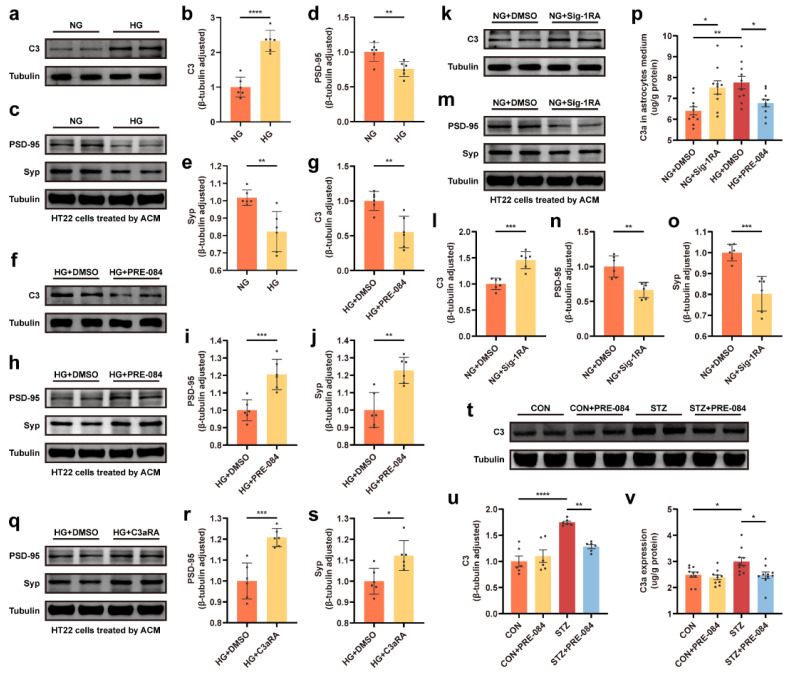
C3/C3a secretion was involved in Sig-1R-regulated synaptic protein loss. (**a**–**e**) Representative Western blot and quantitative analyses of C3 and synaptic protein (PSD-95 and Syp) expression in the NG and HG groups (*n* = 6). (**f**–**j**) Representative Western blot and relevant quantitation analyses of C3 and synaptic protein expression in the HG + DMSO and HG + PRE-084 groups (*n* = 6). (**k**–**o**) Representative Western blot images for C3, PSD-95, and Syp proteins and corresponding quantitative analysis in the NG + DMSO and NG + Sig-1RA groups (*n* = 6). (**p**) Level of C3a secretion in the astrocyte medium measured using ELISA (*n* =10; 5 cell dishes per group). (**q**–**s**) Representative Western blot and relevant quantitation analyses of PSD-95 and Syp protein expression in the HG + DMSO and HG + C3aRA groups (*n* = 6). (**t**,**u**) Representative Western blot analysis for C3 protein expression and relevant quantitation in the CON, CON + PRE-084, STZ, and STZ + PRE-084 groups (*n* = 6). (**v**) Level of C3a production in the hippocampus of normal and diabetic mice with or without PRE-084 treatment using ELISA (*n* = 10; 5 mice per group). The data were presented as mean ± SEM and analyzed using the Student’s *t* test or one-way ANOVA with Tukey’s multiple comparison tests. * *p* < 0.05; ** *p* < 0.01; *** *p* < 0.001; **** *p* < 0.0001. C3/C3a, complement component 3/3a; Sig-1R, sigma-1 receptor; PSD, postsynaptic density; Syp, synaptophysin; NG, normal glucose; HG, high glucose; Sig-1RA, Sig-1R antagonist; C3aRA, C3a receptor antagonist; CON, control mice; STZ, mice with T1DM.

We observed, via Western blotting and ELISA, that the astrocytic C3 protein level and extracellular C3a content were obviously elevated in the HG groups (Figure 5a,b,p). We also observed that the expression of PSD-95 and Syp reduced under HG-ACM incubation compared to NG-ACM incubation (Figure 5c–e). Besides lowering ERS-related protein expression, the Sig-1R agonist also contributed to a decrease in both C3 and C3a production (Figure 5f,g,p). Western blotting demonstrated that the downregulation of PSD-95 and Syp protein levels in HG-ACM was attenuated by treatment with PRE-084 (Figure 5h–j). Moreover, C3 and C3a expression was increased in the NG + Sig-1RA group compared with the astrocytes incubated in the NG group (Figure 5k,l,p), and synaptic protein concentration was reduced after Sig-1RA treatment (Figure 5m–o). We used C3aRA (a selective, high-affinity competitive antagonist of C3aR) to block the binding of C3a in astrocytes and C3aR in neurons, and measured the synaptic protein level to investigate the involvement of C3/C3a release from astrocytes in synaptic protein loss. Treatment with C3aRA recovered synaptic deficits in HG-ACM (Figure 5q–s). We also examined, using Western blot and ELISA techniques, the increase in C3/C3a production in the STZ group than in the CON group, which reduced in the STZ + PRE-084 mice (Figure 5t–v). These findings demonstrated that astrocytic Sig-1R regulated C3/C3a synthesis and secretion and further mediated synaptic loss.

### 3.5. The C3aR Antagonist Improved Synaptic Damage and Cognitive Impairment in Mice with T1DM

**Figure 6:**
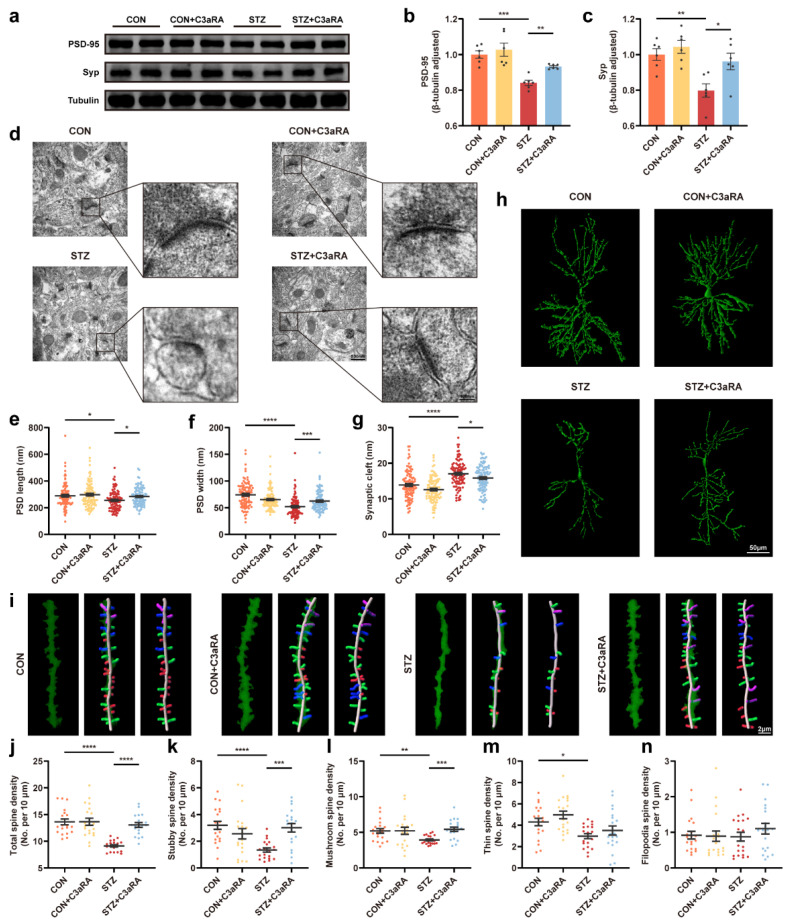
The effect of C3aRA on synapse change in the hippocampus of mice with T1DM. (**a**–**c**) Representative Western blot images and quantitative analysis of synaptic protein expression in CON, CON + C3aRA, STZ, and STZ + C3aRA mice (*n* = 6). (**d**–**g**) Representative ultrastructure images of synapses (**d**) and quantitation of PSD length (**e**) and width (**f**) and synaptic cleft (**g**) (*n* = 95–99; 3 mice per group). The scale bars are 500 and 100 nm. (**h**) Golgi staining images of the neuronal morphology in different groups. The scale bar is 50 μm. (**i**) Representative three-dimensional reconstruction images of different types of hippocampal spines in each group, including stubby (red), mushroom (green), thin (blue), and filopodia (purple) spines. The scale bar is 2 μm. (**j**–**n**) Quantitative analysis of spine density, including total (**j**), stubby (**k**), mushroom (**l**), thin (**m**), and filopodia (**n**) spines (*n* = 20; 3 mice per group). The data were presented as mean ± SEM and analyzed via one-way ANOVA with Tukey’s multiple comparison tests, Welch’s ANOVA with Dunnett’s T3 multiple comparison tests, and Kruskal-Wallis test with Dunn’s multiple comparison tests. * *p* < 0.05; ** *p* < 0.01; *** *p* < 0.001; **** *p* < 0.0001. C3aRA, complement component 3a receptor antagonist; T1DM, type 1 diabetes mellitus; CON, control mice; STZ, mice with T1DM; PSD, postsynaptic density; Syp, synaptophysin.

**Figure 7:**
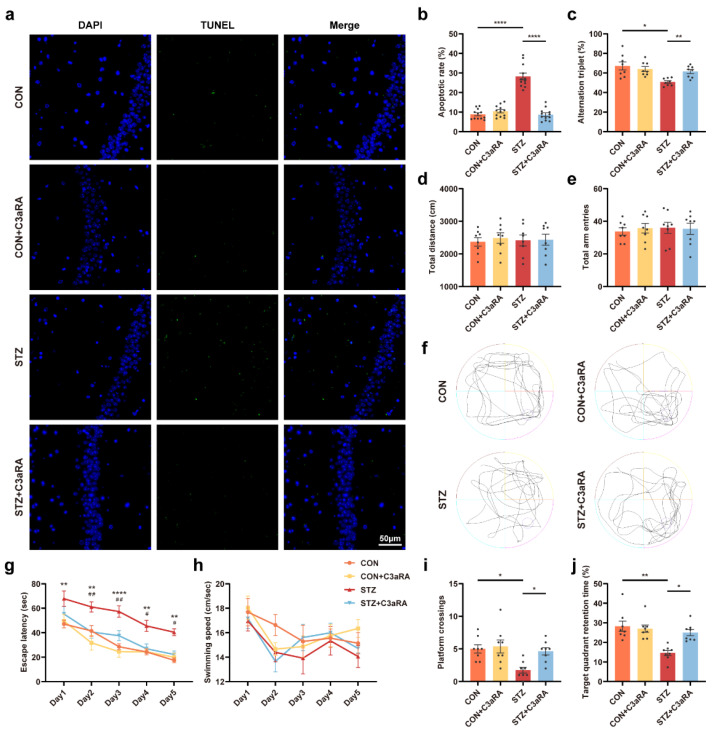
The effect of C3aRA on cognitive function in vivo. (**a**,**b**) Representative images and quantitative analysis of TUNEL labeling of the hippocampus in each group (*n* = 12; 3 mice per group). The scale bar is 50 μm. (**c**–**e**) Y-maze alternation triplet (%) (**c**), total distance (**d**), and total arm entries (**e**) (*n* = 8 mice for all groups). (**f**–**j**) Representative traces for MWM test (**f**) and the quantitation of escape latency (**g**), swimming speed (**h**), platform crossings (**i**), and target quadrant retention time (**j**) (*n* = 8) (* CON vs. STZ, ^#^ STZ vs. STZ + C3aRA). The data were presented as mean ± SEM and analyzed via one-way or two-way ANOVA with Tukey’s multiple comparison tests, Welch’s ANOVA test followed by Dunnett’s T3 multiple comparison tests or Kruskal-Wallis test with Dunn’s multiple comparison tests. * *p* < 0.05; ** *p* < 0.01; **** *p* < 0.0001; ^#^ *p* < 0.05; ^##^ *p* < 0.01. CON, control mice; STZ, mice with T1DM; MWM, Morris water maze; TUNEL, terminal deoxynucleotidyl transferase-mediated dUTP nick-end labeling. C3aRA, complement component 3a receptor antagonist; TUNEL, terminal deoxynucleotidyl transferase-mediated dUTP nick-end labeling; MWM, Morris water maze; T1DM, type 1 diabetes mellitus; CON, control mice; STZ, mice with T1DM.

C3aRA was used in vivo to confirm the complement effect on neurological changes to further determine whether Sig-1R activation-mediated synaptic improvement was due to a decrease in C3/C3a level in the brain of mice with T1DM. Consistent with the aforementioned observations in vitro, the Western blot images exhibited that C3aRA improved the decrease in PSD-95 and Syp protein levels in the STZ group (Figure 6a–c). An amelioration of PSD length and thickness was observed in mice with T1DM treated with C3aRA compared with those in the STZ group (Figure 6d–f). Meanwhile, C3aRA obviously suppressed STZ-induced widening of the synaptic cleft (Figure 6g) and elevated neuronal complexity as well as dendritic length (Figure 6h). The densities of total, stubby, and mushroom spines were significantly improved in mice with T1DM using C3aRA injection (Figure 6i–l). However, the thin and filopodia spines displayed no statistically significant differences between the STZ and STZ + C3aRA groups (Figure 6m,n). There were no statistical differences in density percentage of different types of spines in each group, except in thin spine density percentage between CON + C3aRA and STZ + C3aRA groups (Figure S3). Moreover, the TUNEL assay showed increased cell apoptosis in mice with T1DM, while C3aRA significantly reduced the number of positive apoptotic cells (Figure 7a,b). Furthermore, in the Y-maze test, C3aRA injection independently elevated the accuracy of spontaneous alternating behavior, and the total distance as well as total arm entry values were not different among the four groups (Figure 7c–e). In MWM tests, the average escape latency was shortened in mice with T1DM treated with C3aRA than in untreated mice with T1DM (Figure 7g). C3aRA treatment caused the extension of target quadrant retention time and markedly increased the number of platform crossings in mice with T1DM (Figure 7f,i,j), indicating that elevated C3/C3a deposition might participate in synaptic degeneration and neurodegenerative changes. Thus, these findings implied that the neuroprotective effect of Sig-1R agonists in T1DM was at least partly attributed to the downregulation of C3/C3a.

## 4. Discussion

**Figure 8:**
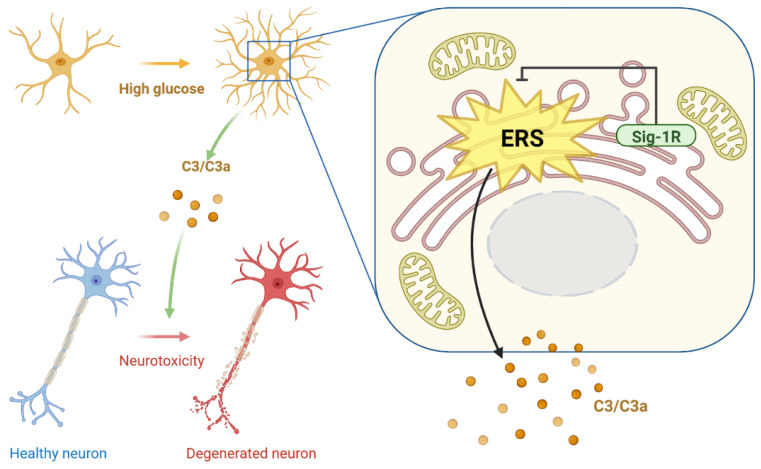
Diagram depicting study mechanism. Activation of astrocytic Sig-1R suppressed HG-induced enhancement in ER-mitochondrion contact, ERS pathway, and C3/C3a production, further restoring damaged neurons. Sig-1R, sigma-1 receptor; HG, high glucose; ER, endoplasmic reticulum; ERS, ER stress; C3/C3a, complement component 3/3a.

DACD has evolved as the second leading contributor to diabetes-caused deaths [2]. As no established treatment exists that can halt or delay DACD progression, except for adequate symptomatic treatments, the underlying mechanism of DACD needs to be elucidated to provide a theoretical framework for its therapy. Sig-1R and C3 are key molecules in regulating neurogenerative diseases. However, their functional relationship with DACD remains unclear. This study demonstrated that HG concentration increased ERS, and the complement cascade reaction might be at least partly regulated by astrocytic Sig-1R. The Sig-1R agonist, PRE-084, reduced astrocytic ER-mitochondrion contact, ERS-related protein expression, and C3/C3a secretion in mice with T1DM. Moreover, the activation of Sig-1R and the inhibition of C3/C3a improved the synaptic and cognitive dysfunctions, providing prospective therapeutic targets for treating DACD (Figure 8).

Sig-1R, as an ER chaperone to bind BIP residing specifically at the lipid raft of MAM, plays a pivotal role in the physiopathology and neuroprotective effects in several neurodegenerative diseases, which deserves translation in preclinical or clinical trials [8]. The number and function of MAM maintain a dynamic and stable balance under normal conditions. However, diseases such as obesity or diabetes drove the enrichment of MAM in various cells, including hepatocytes [34], cardiomyocytes [35], and oocytes [36], resulting in mitochondrial dysfunction, calcium overload, and insulin resistance. In addition to excessive formation, structural destruction of MAMs will also disturb cellular function. Studies have shown that Sig-1R could promote the stabilization and formation of lipid microdomains in ER membrane, and MAM deficiency was observed in Sig-1R knockout mice [9,37]. Consistent with the evidence that MAM alterations and ERS are common features among various neurodegenerative disorders, studies have observed that Sig-1R appeared to induce overt cell homeostasis primarily by directly preventing the occurrence of ERS as well as inflammation [38,39]. In respect of the central nervous system, Sig-1Rs were widely expressed and were detected in neurons, interneurons, and all glial cell types, including astrocytes and microglia. In the hippocampus, Sig-1R presented the ring-like structure staining in astrocytes with glial fibrillary acidic protein immunostaining [40]. The activation of Sig-1R may block the inflammatory response in rodent models of stroke and amyotrophic lateral sclerosis via decreasing the number of reactive astrocytes [13,14], and suppress amyloid-β-mediated hippocampal astrocyte and microglial proliferation [41]. Additionally, elevated astrocytic marker glial fibrillary acidic protein expression caused by Sig-1R deficiency was detected [42]. However, a study found that the deficiency of Sig-1R or its antagonist NE100 repressed astrocyte activation in a Parkinson’s disease model [43]. In addition, methamphetamine induced astrocyte activation through a positive-feedback regulation of Sig-1R expression, thereby contributing to neuroinflammation [44]. The discrepancy in Sig-1R effects in astrocytes may be explained via different models and ligands, but further studies need to confirm this hypothesis. In this study, HG-induced astrocytic ERS was ameliorated after PRE-084 treatment. On the contrary, the Sig-1R antagonist destroyed the stability of ER in NG-cultured astrocytes, which was detrimental to subsequent neuron survival. Hence, the role of Sig-1R is crucial in regulating ERS in astrocytes under high-glucose conditions. Moreover, the increased ER-mitochondria contacts and the decreased distance between them in astrocytes was observed in the diabetic mice, which were rescued by Sig-1R agonist. Taken together, activation of Sig-1R can reduce ER-mitochondrion contact and alleviate ERS in astrocytes of the mice with T1DM.

Sig-1R levels are downregulated in the central nervous system of patients with Alzheimer’s [45,46] and Parkinson’s diseases [47]. Sig-1R agonists could decrease the proportion of Sig-1R multimers formed and induce lipid microdomain remodeling in the ER membrane, which might lead to an improvement in MAM function in neurons of Alzheimer’s disease and amyotrophic lateral sclerosis [37]. Indeed, our results showed that the protein level of Sig-1R decreased in the mice with T1DM or HG-incubated astrocytes but had no obvious change after treatment with the Sig-1R agonist, which might be interpreted as the Sig-1R agonist can only change their conformation and distribution. Evidence suggested that Sig-1R agonists, especially PRE-084, played a protective role against neurodegenerative diseases in a variety of models [10,38]. Studies showed that the Sig-1R ablation mice that exhibited a decrease in motor coordination upregulated p-eIF2α and CHOP expression [48]. Sig-1R knockout or Sig-1R blockade treatment worsened neurotoxicity and behavioral deficits [49], while several agonists, including PRE-084, exhibited neuroprotection in animal models of Parkinson’s [50] and Alzheimer’s diseases [41,51,52,53,54]. Sig-1R stimulation could reduce neuronal amyloid-β deposition [55,56,57] and improve the stability of mushroom spines [58]. Consistent with the findings of previous studies, the spatial learning and memory functions were recovered in the mice with T1DM treated with PRE-084 than in the mice with T1DM in this study, indicating that activating Sig-1R had beneficial effects in DACD.

Evidence indicates that the complement cascade, as one of the primary upregulated inflammatory pathways, appears consistently in various neurodegenerative disorders, driving the degeneration progression and cognitive impairment [22]. In tunicamycin-treated acute ERS models, the complement system activation product C3 was elevated [21]. Astrocytes regulated C3 secretion via the NF-κB signaling pathway, which was closely related to ERS [23,59]. Due to the regulation of Sig-1R on ERS and the destructive effect of the astrocytic C3 secretion on dendritic morphology and neuronal functions [23,24], the improvement in the cognitive function in mice with T1DM using Sig-1R agonists might be partially due to the lack of C3/C3a release. These findings were supported by the results that C3- [27,28] and C3aR-deficient [18] mice were protected against synapse loss as well as a cognitive disorder. Additionally, data from human post-mortem tissue samples confirmed that C3-positive reactive astrocytes were implicated in several neurodegenerative disorders [26,27]. Considering that astrocytes were the crucial source of C3 in the brain, the level of C3/C3a was evaluated under the condition of Sig-1R activation to explore whether C3/C3a secretion was regulated by Sig-1R in vivo. Although the complexity and crosstalk between various immune inflammation pathways may be involved in DACD, this study showed that Sig-1R agonists suppressed C3/C3a production in HG-induced astrocytes and the hippocampus of mice with T1DM. A previous study showed that among the 233 specific proteins interacting with Sig-1R, BIP was specifically enriched [60]. Under pathological conditions, BIP dissociates from the three ERS sensors and activate them to induce unfolded protein reaction and ERS [38]. Another study also detected more than 200 proteins adjacent to Sig-1R at MAMs, which could enrich to “response to ERS” in GO analysis. Sig-1R was in close proximity to complement factor H, which was involved in the complement cascade reaction, but not to C3/C3a directly [9]. Therefore, we speculate that activating Sig-1R may regulate C3 secretion by interacting with complement factor H or ERS-related proteins. Simultaneously, Sig-1R agonists alleviated spine deficits, reduced neuronal apoptosis, and improved cognitive function in mice with T1DM. We examined whether C3aRA pharmacological treatment influenced behavioral outcomes to confirm whether the reduction in C3/C3a, secreted by astrocytes, led to the beneficial effect of Sig-1R agonists as C3/C3a belonged primarily to astrocytes [22]. Consistent with our hypothesis, C3aRA was not only neuroprotective in synapses but also restored neuronal apoptosis and learning memory disabilities in mice with T1DM. Thus, the decrease in mediator C3 production might account for the neuroprotective effects of activating Sig-1R in the mice with T1DM. Although this study showed that the total spine density and several types of the spine in mice with T1DM exhibited stably obvious differences, the thin and filopodia spines displayed no statistical differences between the T1DM and drug treatment groups. This might be because the thin and filopodia spines were unstable and could be formed or removed within hours, while other types of spine remained relatively stable for months or even years [61]. Additionally, we counted the density percentage of different types of spines. There was almost no significant difference among different groups, indicating no changes in the distribution of the different spines. We considered that the difference of thin spines between CON + C3aRA and STZ + C3aRA groups was not representative due to the instability of them. Thus, the absolute numbers and relative percentage of different spines demonstrated that the spine density decreased overall and uniformly in the mice with T1DM, rather than the obvious changes of one or several different types of spines, and the improvement effect of Sig-1R agonists and C3a antagonist was overall. Hence, Sig-1R agonist exhibited neurorestorative effects by reducing astrocytic ER-mitochondrion contact, ERS activation, and complement cascade reaction in DACD.

Our study had several limitations. First, Sig-1Rs are highly expressed in the nervous system and some secretory organs, including the liver [37]. We activated Sig-1R through intraperitoneal injection of its agonist in this study, so we could not exclude the effects of other organs on cognitive function, such as the existence of a liver-brain axis. Since Sig-1Rs were widely expressed in neurons, astrocytes, and microglia in the central nervous system [40], the improvement of cognitive function might not only due to the activation of astrocytic Sig-1R. Second, although we speculate there was an indirect relationship between Sig-1R and C3, it was unclear whether there was a direct protein interaction between Sig-1R and C3. Further research should be performed to investigate such hypotheses and their underlying mechanisms. Currently, a majority of small druggable molecules could activate Sig-1R to be effective neuroprotectants for antipsychotic and neurodegeneration [9,10,62]. For example, pridopidine, the selective Sig-1R agonist, displayed potential efficacy in amyotrophic lateral sclerosis, Alzheimer’s, and Huntington’s disease preclinical or clinical studies [63,64]. Hence, despite the limitations of our study, our research provided further evidence that activation of Sig-1R may be a prospective strategy for DACD treatment.

In conclusion, we revealed that the activation of Sig-1R alleviated the enhancement of ER-mitochondrion contact, ERS activation, and complement cascade reaction in astrocytes, which was advantageous to synaptic and cognitive function recovery. The results of this study not only confirmed the cellular mechanisms of astrocytic Sig-1R activation ameliorating complement-mediated synapse deficits and cognitive disorder in the pathogenesis of DACD, but also provided evidence for ideal clinical translational treatment using Sig-1R agonists.

## Data Availability

Not applicable.

## Supplementary Materials

The following supporting information can be downloaded at: https://www.mdpi.com/article/10.3390/cells12010197/s1. Figure S1: The role of the Sig-1R agonist on ER-mitochondrion contact in the astrocytes of mice with T1DM. Figure S2: The effect of the Sig-1R agonist on different types of spine density percentage. Figure S3: The role of C3aRA on different types of the spine density percentage.

## References

[1] Gao Y., Xiao Y., Miao R., Zhao J., Cui M., Huang G., Fei M. (2016). The prevalence of mild cognitive impairment with type 2 diabetes mellitus among elderly people in China: A cross-sectional study. Arch. Gerontol. Geriatr..

[2] Pearson-Stuttard J., Bennett J., Cheng Y.J., Vamos E.P., Cross A.J., Ezzati M., Gregg E.W. (2021). Trends in predominant causes of death in individuals with and without diabetes in England from 2001 to 2018: An epidemiological analysis of linked primary care records. Lancet Diabetes Endocrinol..

[3] Liu C., Yuan Y.C., Guo M.N., Xin Z., Chen G.J., Bentley A.R., Hua L., Zheng J.P., Ekoru K., Yang J.K. (2021). Incidence of Type 1 Diabetes May Be Underestimated in the Chinese Population: Evidence From 21.7 Million People Between 2007 and 2017. Diabetes Care.

[4] Smolina K., Wotton C.J., Goldacre M.J. (2015). Risk of dementia in patients hospitalised with type 1 and type 2 diabetes in England, 1998-2011: A retrospective national record linkage cohort study. Diabetologia.

[5] Hetz C., Saxena S. (2017). ER stress and the unfolded protein response in neurodegeneration. Nat. Rev. Neurol..

[6] Hetz C., Mollereau B. (2014). Disturbance of endoplasmic reticulum proteostasis in neurodegenerative diseases. Nat. Rev. Neurosci..

[7] Hotamisligil G.S. (2010). Endoplasmic reticulum stress and the inflammatory basis of metabolic disease. Cell.

[8] Wu N.H., Ye Y., Wan B.B., Yu Y.D., Liu C., Chen Q.J. (2021). Emerging Benefits: Pathophysiological Functions and Target Drugs of the Sigma-1 Receptor in Neurodegenerative Diseases. Mol. Neurobiol..

[9] Zhemkov V., Geva M., Hayden M.R., Bezprozvanny I. (2021). Sigma-1 Receptor (S1R) Interaction with Cholesterol: Mechanisms of S1R Activation and Its Role in Neurodegenerative Diseases. Int. J. Mol. Sci..

[10] Penke B., Fulop L., Szucs M., Frecska E. (2018). The Role of Sigma-1 Receptor, an Intracellular Chaperone in Neurodegenerative Diseases. Curr. Neuropharmacol..

[11] Jia J., Cheng J., Wang C., Zhen X. (2018). Sigma-1 Receptor-Modulated Neuroinflammation in Neurological Diseases. Front. Cell. Neurosci..

[12] Tsai S.Y., Pokrass M.J., Klauer N.R., De Credico N.E., Su T.P. (2014). Sigma-1 receptor chaperones in neurodegenerative and psychiatric disorders. Expert Opin. Ther. Targets.

[13] Ajmo C.T., Vernon D.O., Collier L., Pennypacker K.R., Cuevas J. (2006). Sigma receptor activation reduces infarct size at 24 hours after permanent middle cerebral artery occlusion in rats. Curr. Neurovasc. Res..

[14] Peviani M., Salvaneschi E., Bontempi L., Petese A., Manzo A., Rossi D., Salmona M., Collina S., Bigini P., Curti D. (2014). Neuroprotective effects of the Sigma-1 receptor (S1R) agonist PRE-084, in a mouse model of motor neuron disease not linked to SOD1 mutation. Neurobiol. Dis..

[15] Engström G., Hedblad B., Eriksson K.F., Janzon L., Lindgärde F. (2005). Complement C3 is a risk factor for the development of diabetes: A population-based cohort study. Diabetes.

[16] Borné Y., Muhammad I.F., Lorés-Motta L., Hedblad B., Nilsson P.M., Melander O., de Jong E.K., Blom A.M., den Hollander A.I., Engström G. (2017). Complement C3 Associates With Incidence of Diabetes, but No Evidence of a Causal Relationship. J. Clin. Endocrinol. Metab..

[17] Biessels G.J., Nobili F., Teunissen C.E., Simó R., Scheltens P. (2020). Understanding multifactorial brain changes in type 2 diabetes: A biomarker perspective. Lancet Neurol..

[18] Litvinchuk A., Wan Y.W., Swartzlander D.B., Chen F., Cole A., Propson N.E., Wang Q., Zhang B., Liu Z., Zheng H. (2018). Complement C3aR Inactivation Attenuates Tau Pathology and Reverses an Immune Network Deregulated in Tauopathy Models and Alzheimer’s Disease. Neuron.

[19] Lian H., Litvinchuk A., Chiang A.C., Aithmitti N., Jankowsky J.L., Zheng H. (2016). Astrocyte-Microglia Cross Talk through Complement Activation Modulates Amyloid Pathology in Mouse Models of Alzheimer’s Disease. J. Neurosci..

[20] Sun W., Li H., Gu J. (2020). Up-regulation of microRNA-574 attenuates lipopolysaccharide- or cecal ligation and puncture-induced sepsis associated with acute lung injury. Cell Biochem. Funct..

[21] Wang X., Xin H., Xing M., Gu X., Hao Y. (2022). Acute Endoplasmic Reticulum Stress Induces Inflammation Reaction, Complement System Activation, and Lipid Metabolism Disorder of Piglet Livers: A Proteomic Approach. Front. Physiol..

[22] Propson N.E., Gedam M., Zheng H. (2021). Complement in Neurologic Disease. Annu. Rev. Pathol..

[23] Lian H., Yang L., Cole A., Sun L., Chiang A.C., Fowler S.W., Shim D.J., Rodriguez-Rivera J., Taglialatela G., Jankowsky J.L. (2015). NFκB-activated astroglial release of complement C3 compromises neuronal morphology and function associated with Alzheimer’s disease. Neuron.

[24] Zhao Y., Luo C., Chen J., Sun Y., Pu D., Lv A., Zhu S., Wu J., Wang M., Zhou J. (2018). High glucose-induced complement component 3 up-regulation via RAGE-p38MAPK-NF-κB signalling in astrocytes: In vivo and in vitro studies. J. Cell. Mol. Med..

[25] Shi Q., Colodner K.J., Matousek S.B., Merry K., Hong S., Kenison J.E., Frost J.L., Le K.X., Li S., Dodart J.C. (2015). Complement C3-Deficient Mice Fail to Display Age-Related Hippocampal Decline. J. Neurosci..

[26] Liddelow S.A., Guttenplan K.A., Clarke L.E., Bennett F.C., Bohlen C.J., Schirmer L., Bennett M.L., Münch A.E., Chung W.S., Peterson T.C. (2017). Neurotoxic reactive astrocytes are induced by activated microglia. Nature.

[27] Wu T., Dejanovic B., Gandham V.D., Gogineni A., Edmonds R., Schauer S., Srinivasan K., Huntley M.A., Wang Y., Wang T.M. (2019). Complement C3 Is Activated in Human AD Brain and Is Required for Neurodegeneration in Mouse Models of Amyloidosis and Tauopathy. Cell Rep..

[28] Shi Q., Chowdhury S., Ma R., Le K.X., Hong S., Caldarone B.J., Stevens B., Lemere C.A. (2017). Complement C3 deficiency protects against neurodegeneration in aged plaque-rich APP/PS1 mice. Sci. Transl. Med..

[29] Rom S., Zuluaga-Ramirez V., Gajghate S., Seliga A., Winfield M., Heldt N.A., Kolpakov M.A., Bashkirova Y.V., Sabri A.K., Persidsky Y. (2019). Hyperglycemia-Driven Neuroinflammation Compromises BBB Leading to Memory Loss in Both Diabetes Mellitus (DM) Type 1 and Type 2 Mouse Models. Mol. Neurobiol..

[30] Watanabe S., Ilieva H., Tamada H., Nomura H., Komine O., Endo F., Jin S., Mancias P., Kiyama H., Yamanaka K. (2016). Mitochondria-associated membrane collapse is a common pathomechanism in SIGMAR1- and SOD1-linked ALS. EMBO Mol. Med..

[31] Stoica R., De Vos K.J., Paillusson S., Mueller S., Sancho R.M., Lau K.F., Vizcay-Barrena G., Lin W.L., Xu Y.F., Lewis J. (2014). ER-mitochondria associations are regulated by the VAPB-PTPIP51 interaction and are disrupted by ALS/FTD-associated TDP-43. Nat. Commun..

[32] Spat A., Szanda G., Csordas G., Hajnoczky G. (2008). High- and low-calcium-dependent mechanisms of mitochondrial calcium signalling. Cell Calcium.

[33] Li Z.Z., Han W.J., Sun Z.C., Chen Y., Sun J.Y., Cai G.H., Liu W.N., Wang T.Z., Xie Y.D., Mao H.H. (2021). Extracellular matrix protein laminin β1 regulates pain sensitivity and anxiodepression-like behaviors in mice. J. Clin. Investig..

[34] Arruda A.P., Pers B.M., Parlakgul G., Guney E., Inouye K., Hotamisligil G.S. (2014). Chronic enrichment of hepatic endoplasmic reticulum-mitochondria contact leads to mitochondrial dysfunction in obesity. Nat. Med..

[35] Wu S., Lu Q., Ding Y., Wu Y., Qiu Y., Wang P., Mao X., Huang K., Xie Z., Zou M.H. (2019). Hyperglycemia-Driven Inhibition of AMP-Activated Protein Kinase alpha2 Induces Diabetic Cardiomyopathy by Promoting Mitochondria-Associated Endoplasmic Reticulum Membranes In Vivo. Circulation.

[36] Zhao L., Lu T., Gao L., Fu X., Zhu S., Hou Y. (2017). Enriched endoplasmic reticulum-mitochondria interactions result in mitochondrial dysfunction and apoptosis in oocytes from obese mice. J. Anim. Sci. Biotechnol..

[37] Zhemkov V., Ditlev J.A., Lee W.R., Wilson M., Liou J., Rosen M.K., Bezprozvanny I. (2021). The role of sigma 1 receptor in organization of endoplasmic reticulum signaling microdomains. eLife.

[38] Delprat B., Crouzier L., Su T.P., Maurice T. (2020). At the Crossing of ER Stress and MAMs: A Key Role of Sigma-1 Receptor?. Adv. Exp. Med. Biol..

[39] Vallese F., Barazzuol L., Maso L., Brini M., Calì T. (2020). ER-Mitochondria Calcium Transfer, Organelle Contacts and Neurodegenerative Diseases. Adv. Exp. Med. Biol..

[40] Liu Q., Guo Q., Fang L.P., Yao H., Scheller A., Kirchhoff F., Huang W. (2022). Specific detection and deletion of the sigma-1 receptor widely expressed in neurons and glial cells in vivo. J. Neurochem..

[41] Maurice T., Volle J.N., Strehaiano M., Crouzier L., Pereira C., Kaloyanov N., Virieux D., Pirat J.L. (2019). Neuroprotection in non-transgenic and transgenic mouse models of Alzheimer’s disease by positive modulation of σ(1) receptors. Pharmacol. Res..

[42] Weng T.Y., Hung D.T., Su T.P., Tsai S.A. (2017). Loss of Sigma-1 Receptor Chaperone Promotes Astrocytosis and Enhances the Nrf2 Antioxidant Defense. Oxid. Med. Cell. Longev..

[43] Hong J., Sha S., Zhou L., Wang C., Yin J., Chen L. (2015). Sigma-1 receptor deficiency reduces MPTP-induced parkinsonism and death of dopaminergic neurons. Cell Death Dis..

[44] Zhang Y., Lv X., Bai Y., Zhu X., Wu X., Chao J., Duan M., Buch S., Chen L., Yao H. (2015). Involvement of sigma-1 receptor in astrocyte activation induced by methamphetamine via up-regulation of its own expression. J. Neuroinflamm..

[45] Mishina M., Ohyama M., Ishii K., Kitamura S., Kimura Y., Oda K., Kawamura K., Sasaki T., Kobayashi S., Katayama Y. (2008). Low density of sigma1 receptors in early Alzheimer’s disease. Ann. Nucl. Med..

[46] Hedskog L., Pinho C.M., Filadi R., Rönnbäck A., Hertwig L., Wiehager B., Larssen P., Gellhaar S., Sandebring A., Westerlund M. (2013). Modulation of the endoplasmic reticulum-mitochondria interface in Alzheimer’s disease and related models. Proc. Natl. Acad. Sci. USA.

[47] Mishina M., Ishiwata K., Ishii K., Kitamura S., Kimura Y., Kawamura K., Oda K., Sasaki T., Sakayori O., Hamamoto M. (2005). Function of sigma1 receptors in Parkinson’s disease. Acta Neurol. Scand..

[48] Hong J., Wang L., Zhang T., Zhang B., Chen L. (2017). Sigma-1 receptor knockout increases α-synuclein aggregation and phosphorylation with loss of dopaminergic neurons in substantia nigra. Neurobiol. Aging.

[49] Maurice T., Strehaiano M., Duhr F., Chevallier N. (2018). Amyloid toxicity is enhanced after pharmacological or genetic invalidation of the σ(1) receptor. Behav. Brain Res..

[50] Francardo V., Bez F., Wieloch T., Nissbrandt H., Ruscher K., Cenci M.A. (2014). Pharmacological stimulation of sigma-1 receptors has neurorestorative effects in experimental parkinsonism. Brain.

[51] Lahmy V., Meunier J., Malmström S., Naert G., Givalois L., Kim S.H., Villard V., Vamvakides A., Maurice T. (2013). Blockade of Tau hyperphosphorylation and Aβ_1–42_ generation by the aminotetrahydrofuran derivative ANAVEX2-73, a mixed muscarinic and σ₁ receptor agonist, in a nontransgenic mouse model of Alzheimer’s disease. Neuropsychopharmacology.

[52] Meunier J., Ieni J., Maurice T. (2006). The anti-amnesic and neuroprotective effects of donepezil against amyloid beta25-35 peptide-induced toxicity in mice involve an interaction with the sigma1 receptor. Br. J. Pharmacol..

[53] Villard V., Espallergues J., Keller E., Alkam T., Nitta A., Yamada K., Nabeshima T., Vamvakides A., Maurice T. (2009). Antiamnesic and neuroprotective effects of the aminotetrahydrofuran derivative ANAVEX1-41 against amyloid beta(25-35)-induced toxicity in mice. Neuropsychopharmacology.

[54] Yang R., Chen L., Wang H., Xu B., Tomimoto H., Chen L. (2012). Anti-amnesic effect of neurosteroid PREGS in Aβ25-35-injected mice through σ1 receptor- and α7nAChR-mediated neuroprotection. Neuropharmacology.

[55] Behensky A.A., Yasny I.E., Shuster A.M., Seredenin S.B., Petrov A.V., Cuevas J. (2013). Afobazole activation of σ-1 receptors modulates neuronal responses to amyloid-β25-35. J. Pharmacol. Exp. Ther..

[56] Marrazzo A., Caraci F., Salinaro E.T., Su T.P., Copani A., Ronsisvalle G. (2005). Neuroprotective effects of sigma-1 receptor agonists against beta-amyloid-induced toxicity. Neuroreport.

[57] Kim W.S., Fu Y., Dobson-Stone C., Hsiao J.T., Shang K., Hallupp M., Schofield P.R., Garner B., Karl T., Kwok J.B.J. (2018). Effect of Fluvoxamine on Amyloid-β Peptide Generation and Memory. J. Alzheimers Dis..

[58] Ryskamp D., Wu L., Wu J., Kim D., Rammes G., Geva M., Hayden M., Bezprozvanny I. (2019). Pridopidine stabilizes mushroom spines in mouse models of Alzheimer’s disease by acting on the sigma-1 receptor. Neurobiol. Dis..

[59] Salminen A., Kaarniranta K., Kauppinen A. (2020). ER stress activates immunosuppressive network: Implications for aging and Alzheimer’s disease. J. Mol. Med..

[60] Zhao J., Veeranan-Karmegam R., Baker F.C., Mysona B.A., Bagchi P., Liu Y., Smith S.B., Gonsalvez G.B., Bollinger K.E. (2022). Defining the Ligand-dependent Interactome of the Sigma 1 Receptor. bioRxiv.

[61] Berry K.P., Nedivi E. (2017). Spine Dynamics: Are They All the Same?. Neuron.

[62] Albayrak Y., Hashimoto K. (2017). Sigma-1 Receptor Agonists and Their Clinical Implications in Neuropsychiatric Disorders. Adv. Exp. Med. Biol..

[63] Ye N., Qin W., Tian S., Xu Q., Wold E.A., Zhou J., Zhen X.C. (2020). Small Molecules Selectively Targeting Sigma-1 Receptor for the Treatment of Neurological Diseases. J. Med. Chem..

[64] Maurice T. (2021). Bi-phasic dose response in the preclinical and clinical developments of sigma-1 receptor ligands for the treatment of neurodegenerative disorders. Expert Opin. Drug Discov..

