# Supplementary material for: Sigma-1 Receptor as a Protective Factor for Diabetes-Associated Cognitive Dysfunction via Regulating Astrocytic Endoplasmic Reticulum-Mitochondrion Contact and Endoplasmic Reticulum Stress
Source: Cells. 2023 Jan 3;12(1):197. doi: 10.3390/cells12010197 (PMC9818229; doi:10.3390/cells12010197)
Supplement: Supplementary file 1 [file cells-12-00197-s001.zip › cells-2062428-supplementary.pdf]

cells MDPI

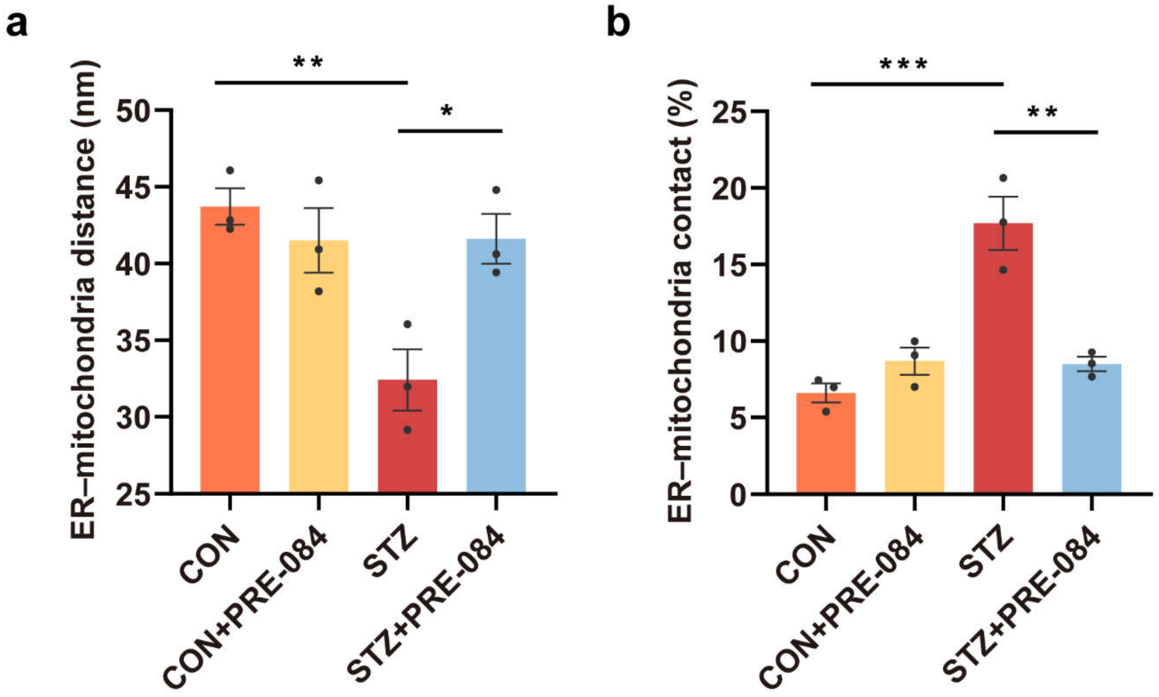

Figure S1. The role of Sig-1R agonist on ER-mitochondrion contact in the astrocytes of mice with T1DM.

(**a**) Quantitative analysis of the distance for ER-mitochondria contacts in astrocytes of CON, CON + PRE-084, STZ, and STZ + PRE-084 mice (n = 3). (**b**) Quantitative analysis of astrocytic ER-mitochondria contact percentage (n = 3). Each point represents the average value of a mouse for corresponding index. The data were presented as mean ± SEM and analyzed using one-way ANOVA with Tukey's multiple comparisons test. * $p < 0.05$; ** $p < 0.01$; *** $p < 0.001$. Sig-1R, sigma-1 receptor; ER, endoplasmic reticulum; T1DM, type 1 diabetes mellitus; CON, control mice; STZ, mice with T1DM.

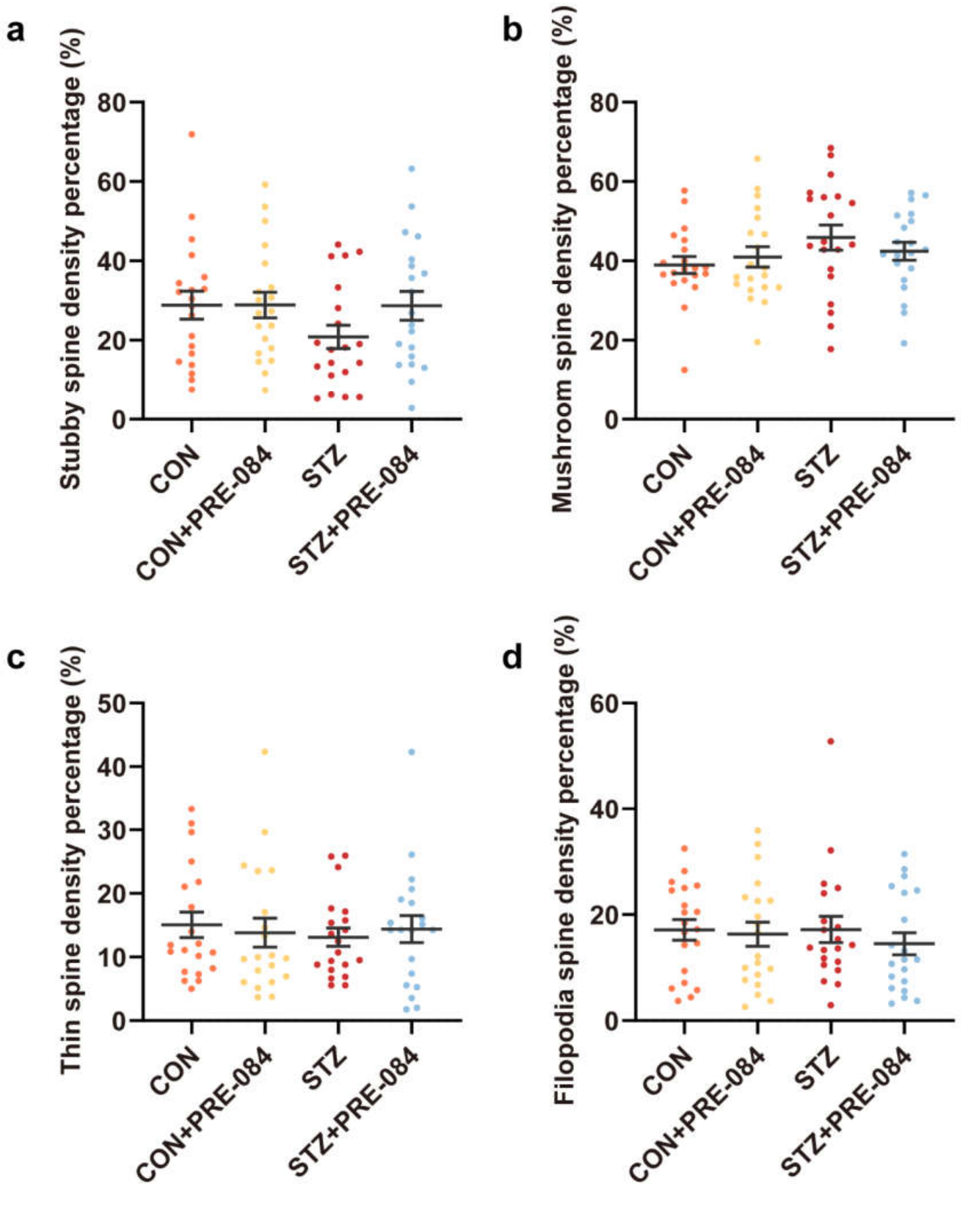

Figure S2. The effect of Sig-1R agonist on different spine density percentage.

(**a**) Quantitative analysis of stubby spine density percentage in CON, CON + PRE-084, STZ, and STZ + PRE-084 mice (n = 20). (**b**) Quantitative analysis of mushroom spine density percentage in each group (n = 20). (**c**) Quantitative analysis of thin spine density percentage in each group (n = 20). (**d**) Quantitative analysis of filopodia spine density percentage in each group (n = 20). The data were presented as mean ± SEM and analyzed via one-way ANOVA with Tukey's multiple comparison tests, and Kruskal-Wallis test followed by Dunn's multiple comparison tests. Sig-1R, sigma-1 receptor; CON, control mice; STZ, mice with type 1 diabetes mellitus.

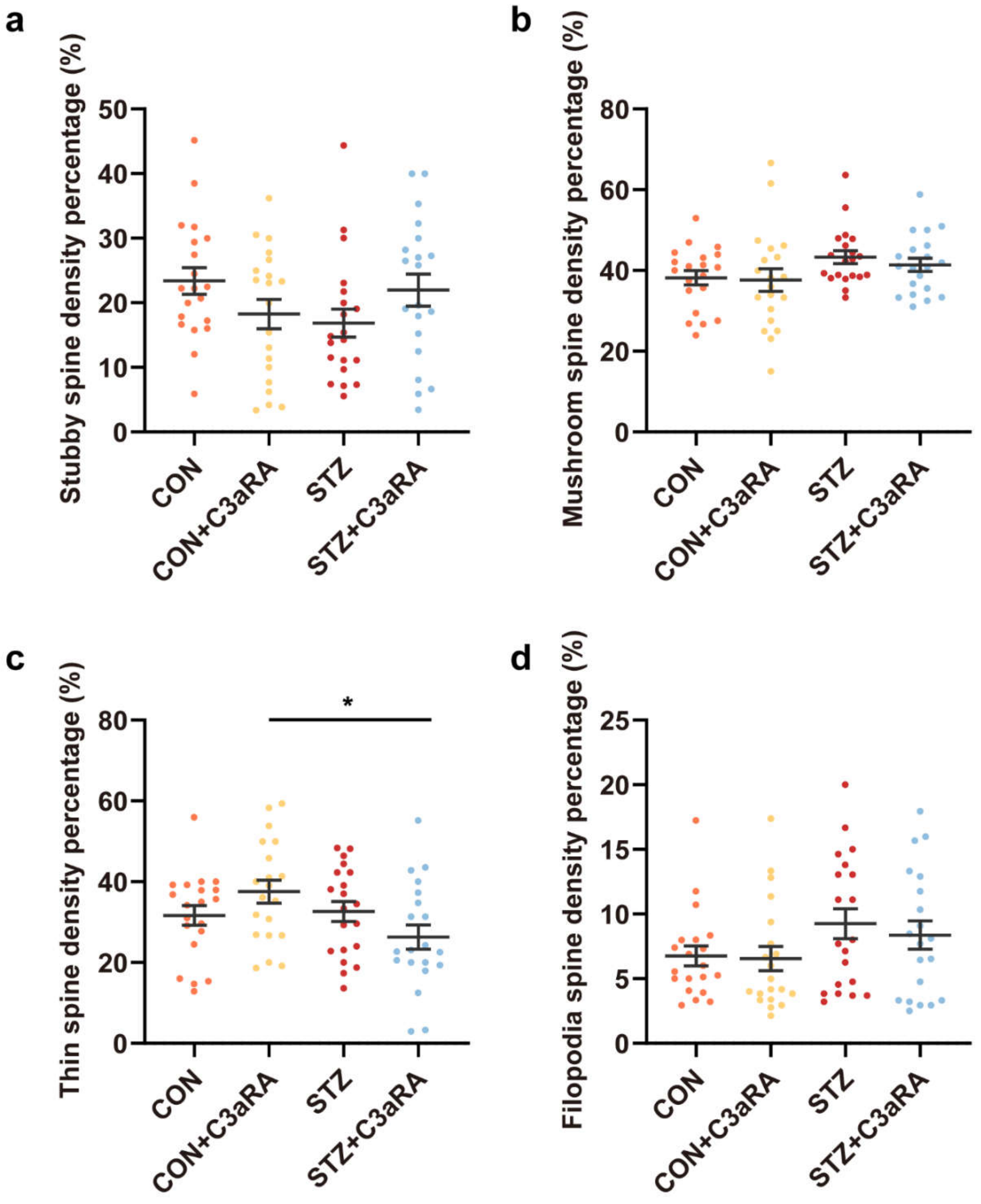

Figure S3. The role of C3aRA on different types of the spine density percentage.

(**a**) Quantitative analysis of stubby spine density percentage in CON, CON + C3aRA, STZ, and STZ + C3aRA mice (n = 20). (**b**) Quantitative analysis of mushroom spine density percentage in each group (n = 20). (**c**) Quantitative analysis of thin spine density percentage in each group (n = 20). (**d**) Quantitative analysis of filopodia spine density percentage in each group (n = 20). The data were presented as mean ± SEM and analyzed via one-way ANOVA with Tukey's multiple comparison tests, and Kruskal-Wallis test followed by Dunn's multiple comparison tests. * $p < 0.05$. C3aRA, complement component 3a receptor antagonist; CON, control mice; STZ, mice with type 1 diabetes mellitus.
